# Supplementary material for: Chromothripsis during telomere crisis is independent of NHEJ, and consistent with a replicative origin
Source: Genome Res. 2019 May;29(5):737–49. doi: 10.1101/gr.240705.118 (PMC6499312; doi:10.1101/gr.240705.118)
Supplement: Supplemental Material [file supp_gr.240705.118_Supplemental_file_1.zip › contigs/annotated_contigs/DB49/contig.2.DB49_length_450_mean_cov_9.99555555556.docx]

**DB49_length_450_mean_cov_9.99555555556**

```
AAC|AAAAAGAAAAAAAAAAAAAAAAAAAAAAAAACAAAAAAAAAAAAAAAAAAAAAAAAAAAAAAAAAAAAAAA|AAAAAAAAAAAAA
    >chr4:19079462-19079533 - E=4e-19 p=0e+00                               >chr8:11879
AAAAAAAAAAAAAATCAGTGAACTTAAAGACATATCAATAAAAATTATCCAAACTGAAATGTAAAAAGAAAAAATCATTTAAAACACAA
9912-118800288 - E=4e-215
AGAGAGCTTCCACAAGAGAAGGATAATATCTAACAGTCTAACTTTAAATAATTAGAGTCCTCCAAAAAAGAGGTTAGAGTGAATAGGGG

AGAAAAAAATATTTGAAGACACAATGGCTGAGAACTTTTCAAAAATGGATGTAATCTTTAGATTAACTCACAAATCCAAGAAACTCAAT

CAATTCCAAACAGGATAAATATGAAGAAAAATATACCTGAGCACAAAATAGTCAAGTTATTGAGAATCAAAGATAAAAAGTGCCCTCTA

AAAATAT
```
